# Public health need versus sales of antibacterial agents active against multidrug-resistant bacteria: a historical perspective

**DOI:** 10.1093/jac/dkt478

**Published:** 2013-12-16

**Authors:** Dominique L. Monnet, Johan Giesecke

**Affiliations:** Office of the Chief Scientist, European Centre for Disease Prevention and Control (ECDC), Tomtebodavägen 11A, SE-171 83 Stockholm, Sweden

**Keywords:** antimicrobials, antibacterials, antibiotics, antimicrobial resistance, multidrug-resistant, market, pharmacoeconomics

Sir,

Anthony R. White, on behalf of the BSAC Working Party on The Urgent Need: Regenerating Antibacterial Drug Discovery and Development, has reviewed the economics of antibacterial resistance and its control.^1^ In this letter, we add to the current knowledge base on this issue by providing a historical perspective on the public health need for antibacterial agents active against emerging multidrug-resistant bacteria and the sales of selected agents, chosen as examples, that responded to this need in previous decades. Data on the worldwide sales of these agents and their dates of approval in the USA were obtained from MedAdNews (http://www.pharmalive.com/med-ad-news), as well as companies’ annual reports (Figure 1).

**Figure 1.**
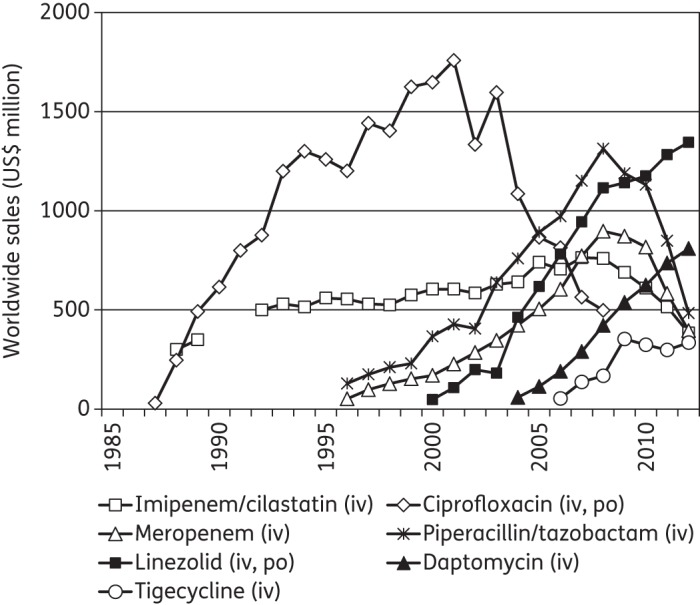
Worldwide sales of patented antibacterial agents chosen as examples, 1985–2012. These agents targeted multidrug-resistant bacteria that were prevalent at the time of their launch. Data do not include sales of generic versions of the same agents marketed after expiry of the patents. The route of administration is indicated in parentheses: iv, intravenous; po, oral. Source: MedAdNews, 1995–2012, and company reports.

In the mid-1980s, the rapid emergence of multidrug-resistant Gram-negative bacteria such as extended-spectrum β-lactamase (ESBL)-producing Enterobacteriaceae^2^ and ceftazidime-resistant *Pseudomonas aeruginosa*^3^ highlighted the need for antibacterial agents active against these resistant bacteria. Imipenem/cilastatin, the first carbapenem, was approved in 1985 and immediately responded to this need. From the mid-1980s into the 1990s, imipenem/cilastatin was used to treat patients infected with such multidrug-resistant Gram-negative bacteria (Figure 1). Meropenem, a second carbapenem, was approved in 1996 and its sales rapidly increased. In 2013, meropenem and imipenem/cilastatin are still essential antibacterial agents for the treatment of healthcare-associated infections, especially in severely ill or immunocompromised patients.^4,5^ Both are classified by the WHO as critically important antimicrobials in human medicine.^6^

During the last two decades, several other antibacterial agents active against emerging multidrug-resistant bacteria, and thus responding to the public health need, have reached substantial sales (Figure 1). Piperacillin/tazobactam, approved in 1993, found its market in the 2000s, in particular as an agent to treat healthcare-associated infections. Linezolid, approved in 2000 as the first oxazolidinone, and daptomycin, approved in 2003 as the first lipopeptide, quickly found their markets as agents to treat infections with Gram-positive bacteria, in particular methicillin-resistant *Staphylococcus aureus*. The sales of linezolid, which is available for both intravenous and oral use, should soon reach a similar level to that of ciprofloxacin in the late 1990s to early 2000s (Figure 1). The only exception to these trends may be tigecycline, approved in 2005 as the first glycylcycline, but did not yet reach annual worldwide sales of US$500 million (Figure 1).

The increasing use of carbapenems, together with the varying quality of infection control practices, has led to the emergence of carbapenem-resistant Gram-negative bacteria. As early as the late 1980s, the percentage of *P. aeruginosa* isolates that were resistant to carbapenems started to increase.^7^ In 2007, the number of carbapenem-resistant *P. aeruginosa* infections in the European Union was estimated at 141 900 annually and the number of multidrug-resistant *P. aeruginosa* infections in the USA at 72 250 annually.^8,9^ More recently, carbapenemase-producing Enterobacteriaceae have emerged and are now spreading globally, including in the European Union.^10–13^ The generally favoured option for the treatment of patients infected by multiresistant carbapenemase-producing bacteria is colistin, an old antibiotic that has now become an essential drug in hospitals. The consequence of the increasing use of colistin, however, is the inevitable rise of colistin resistance, with high percentages of resistance already occurring in *K. pneumoniae* infections in some European hospitals,^14,15^ leading to clinical situations where there is no straightforward option for treatment.

In 2009, a joint report from the European Centre for Disease Prevention and Control and the European Medicines Agency, in a collaboration with ReAct—Action on Antimicrobial Resistance, highlighted the gap between increasing antimicrobial resistance in Europe and the urgent need for new antibacterial agents active against resistant bacteria, in particular to treat multidrug-resistant Gram-negative infections.^8^ Although comparable to the need for agents to treat patients with multidrug-resistant Gram-negative infections (in particular those caused by ESBL-producing bacteria) in the mid-1980s and the 1990s, the current public health need for new antibacterial agents is still currently not being addressed. The succession of cycles of public health need followed by antibacterial productivity, as illustrated by White,^1^ that prevailed since the discovery of antibiotics seems to have come to a halt. Two recent reviews of the antibacterial research and development pipeline confirm that, despite some progress, there are currently only a few compounds in development with activity against multidrug-resistant Gram-negative bacteria, in particular metallo-carbapenemase (e.g. New Delhi metallo-β-lactamase)-producing Enterobacteriaceae or *P. aeruginosa*, or against multidrug-resistant *Acinetobacter* spp.^16,17^

This historical perspective shows that, in the presence of a public health need, antibacterial agents that targeted emerging multidrug-resistant bacteria quickly found their market and reached annual worldwide sales of US$500 million or above, sometimes reaching the ‘blockbuster’ threshold of US$1 billion for worldwide sales annually before the 10th full year after their launch (Figure 1). This observation goes against the common view that new antibacterial agents have a small potential market of less than US$500 million annually.^18^ It provides an insight into what would be the potential market, considering the current public health need and current economic model,^1^ for a novel antibacterial agent active against carbapenem-resistant Gram-negative bacteria.

## Funding

This study was conducted as part of our routine work.

## Transparency declarations

None to declare.
